# Informal healthcare sector and marginalized groups: Repeat visits in rural North India

**DOI:** 10.1371/journal.pone.0199380

**Published:** 2018-07-06

**Authors:** Richard A. Iles

**Affiliations:** Washington State University, Pullman, Washington, United States of America; Indian Institute of Science, INDIA

## Abstract

The interrelationship between the public and private sectors, and formal and informal healthcare sectors effects market-level service quality, pricing behaviour and referral networks. However, health utilisation analysis of national survey data from many low and middle income countries is constrained by the lack of disaggregated health provider data. This study is concerned with the pattern of repeat outpatient consultations for a single episode of fever from public and private qualified providers and private unqualified providers. Cross-sectional survey data from 1173 adult respondents sampled from three districts within India’s most populous state—Uttar Pradesh is analysed. Data was collected during the monsoon season—September to October—in 2012. Regression analysis focuses on the pattern of repeats visits for a single episode of mild-sever fever as the dependent variable. Results show that Women and Muslims in rural north India are more likely to not access healthcare, and if they do, consult with low quality unqualified outpatient healthcare providers. For fever durations of four or more days, men are more likely to access unqualified providers compared to women. Results of the current study supports the literature that women’s utilisation of outpatient healthcare for communicable illnesses in LMICs is often less than men. A relative lack of access to household resources explains why fever duration parameter estimates for women and men differ.

## Introduction

The outcome of health systems is a central policy focus in many low and middle countries (LMIC) as governments strive to provide universal health coverage. In many LMIC contexts, the further development of health systems to achieve strong health and societal goals will require regulatory reform of current institutions, policies and mechanisms.[1] However, in light of the limited data and empirical analysis concerning healthcare provider behaviour in the formal and informal sectors, patients’ heterogeneous preferences and social structures, further analysis of the health systems in LMIC is required before constructive, progressive regulatory reform is possible.

The interrelationship between provider and patient behaviours is central to understanding any health system. Knowledge of this relationship is particularly important in LMIC where regulatory regimes are often weak. Limited regulatory oversight and enforcement helps stimulate ‘innovative’ and often unintended policy results.[2] While the policy reform need is great, the limited available data from LMIC makes the task of building required policy knowledge is challenging.[3] This challenge is exemplified with respect to the activities of unqualified allopathic (i.e. ‘western medicine’) providers who operate in the informal healthcare sector.

The informal healthcare sector is a common feature of many developing economies.[2, 4–7] The informal healthcare sector is defined as producers of goods and services that are not State authorised or registered. This definition reflects that given by Bloom and colleagues[8] who state that the informal sector is defined by the residual of activities that are ‘recognised in law or by legally recognised regulatory agencies…’. In north India, Pinto[9] aptly located unqualified outpatient providers as operating on the “margins of legitimacy”. Despite this high level of illegitimacy, estimates of the market share of the informal outpatient health sector in India range from 48 percent to 80 percent.[10–12]

Estimates of the market share held by the informal healthcare sector in north India rely on non-government data. The highly utilised household surveys, such as the Demographic and Health Survey and National Sample Survey Organisation, provide aggregated healthcare utilisation for all private providers. The aggregation of private providers includes unqualified providers, small clinics operating ‘western’ and/or Indian systems of medicine and large multi-speciality tertiary facilities. As a result, disaggregated healthcare utilisation analysis of marginalised and non-marginalised groups, by provider type, is limited. [13–15] The limited available policy insight into the utilisation of healthcare services by marginalised groups, particularly women and Muslims, in the context of health system reforms risks further disempowerment of these groups.

Counterfactual estimates of the informal outpatient sector indicate that this dominant sector is likely to continue to play a significant role in outpatient service provision to north India’s predominantly rural population. Such estimates, based on the assumption that government doctor absenteeism was eliminated, the informal outpatient sector would reduce from approximately 60% market share to 34% for fever treatments, with the public sector expanding by the same proportion.[12] However, given the current market share estimates of the informal outpatient sector and counterfactual estimates to its continued importance, the role of this sector in universal healthcare coverage in LMICs is unclear. As a consequence, further policy relevant data and analysis is required to inform policy development.

The central objective of this study is to identify patterns of outpatient utilisation across repeat consultations for a single episode of fever. The first specific objective is to compare the patterns of utilisation across qualified and unqualified providers. A second specific objective is to identify the patterns of utilisation among women and Muslims, as marginalised social groups, in rural North India.

## Methods

### Study design

This study uses cross-sectional self-reported survey data collected from a sample of 1173 adults (aged ≥ 18 years). The recall period for this study is 12-months (see Text A in S1 Supporting Information for details of shorter recall periods). The data was collected by the author across three districts (out of a total of 71 districts) of India’s most populous state—Uttar Pradesh. The data was collected between September and October 2012. Copies of the survey tools are available. [16] Respondents were surveyed once and asked whether they made more than one visit to outpatient healthcare providers for a given episode of fever. If so, respondents were asked to indicate if the same or different providers were consulted. Districts were selected due to their representative mean income profiles and for accessibility reasons.[17] The district mean incomes of the three districts cover the interquartile range of Uttar Pradesh.

The sampling units used in this study were district level development blocks. These were selected at random. At the tertiary level, village administrative units from the select blocks were stratified according to the Hindu and Muslim religious majority. Assistance in stratifying was obtained from district Block Development Offices. In total, eight administrative units were selected: four from Fatehpur district and two each from Lalitpur and Balrampur districts. Fatehpur is located in UP’s central region. Proportional sampling of Hindu and Muslim individuals (three Hindu majority administrative units and one Muslim majority) reflecting state-level proportions. The small proportion of Muslim residents in Lalitpur, in the Bundelkhand region, limited the ability to sample Muslim respondents. This was balanced by sampling an equal number of administrative units in Balrampur, which had a strong Muslim representation, and is located in the Uttar Pradesh’s eastern region.

The use of single episode repeat visit utilisation data is unique within quantitative analysis. The desirability of using this data to analyse outpatient utilisation is founded on two reasons: market realities of low regulatory healthcare environment and dual dimensionality. The power and informational imbalance between healthcare providers and patients (principle—agent paradigm), particularly in markets with limited effective regulation of healthcare provider behaviours, suggests that concerns about supplier-induced demand and low healthcare provider effort is a likely problem. [10] The potential interaction of these two effects is a topic of further research. The dual dimensions of initial provide choice and the sequence of follow-up consultations provides more information about potential interactions between provider and patient behaviours.

Institutional and local community ethics approval for this research were provided by Griffith University (Australia) Human Research Ethics Committee (Protocol Number: AFE/07/11/HREC) and local elected village administrative leaders within the respective communities. Informed verbal consent was obtained by all respondents prior to survey administration. In-country institutional ethics approval was not required by the Human Research Ethics Committee for survey work in India. Moreover, the Indian host research institution did not have a human ethics committee at the time of the study. No individually identifiable information was collected or used during analysis.

### Statistical analysis

**Fig 1:**
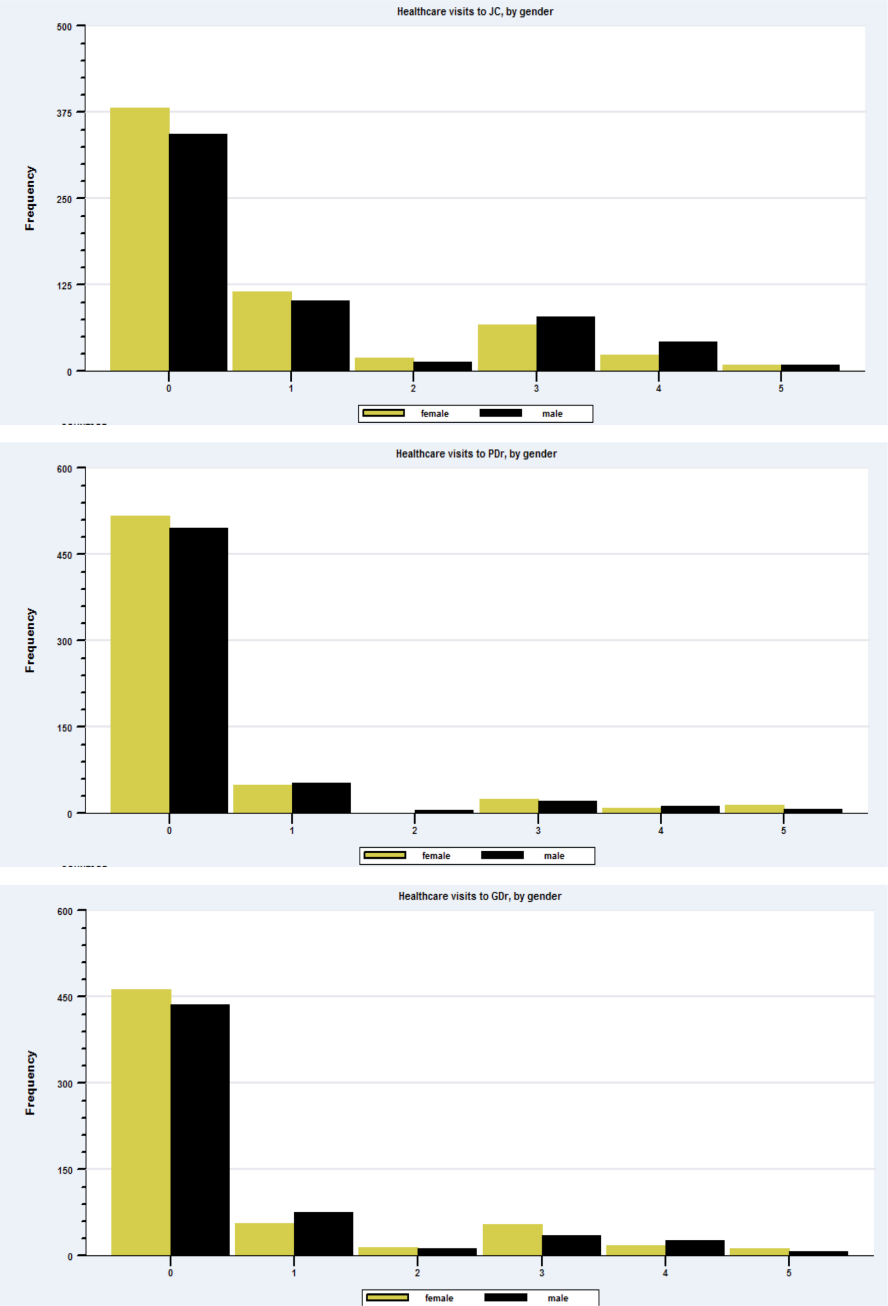
Frequency distribution of visits to healthcare providers for a single fever episode over 12-months.

The zero-inflated ordered probit (ZIOP) model proposed by Harris and Zhao[18] incorporates a latent binary probit regression that separates fever patients from non-fever patients and an ordered probit model.[18] Accounting for two separate processes that explain observed zero consultations, via latent classes, has the effect of inflating the estimated number of zeros. Harris and Zhao[18] explains the zero-inflated component of the model: “the probability of a zero observation has been inflated as it is a combination of the probability of ‘zero-consumption’ from the OP [Ordered Probit] process plus the probability of ‘non-participation’…”.[18] The application of standard cardinal count models are not appropriate in this study due to the bimodal distribution of the dependent variable for visits greater than zero (see Fig 1) and the incorrect assumption that doubling the number of healthcare provider visits equates to a doubling in the amount of healthcare received. [19, 20] A strong of body of literature indicates that outpatient healthcare provider effort should not be assumed to be consistent across time or across provider types (private vs public or qualified vs unqualified). [10, 21] As a result, the ordered probit model is preferred because it imposes no distributional assumptions over the number of reported consultations for a single episode of fever.

These estimates are each carried out using the software *Limdep* version 10. The left-hand side dependent variable is the number of healthcare provider visits made for a single episode of fever. The functional forms for the ZIOP estimates are outlined in S1 Supporting Information (see Text B in S1 Supporting Information). The parameter estimates for the OP and ZIOP models have no direct interpretation. Instead, the marginal effects for each discrete dependent variable values are presented below. Marginal effects estimates reflect the incremental probability for variables as the dependent variables increase in value by one unit. Estimates of boundary (threshold) parameters and goodness-of-fit measures for provider type specific OP and ZIOP estimates for model 1 are presented in S1 Supporting Information (see Table C in S1 Supporting Information).

### Health context and variables

Life-cycle approaches to healthcare demand theory help to explain the complex interpersonal dynamics of health capital decisions within households. Viewing intra-household decision-making in a bargaining framework is common. [22–25] The respective bargaining power between spouses influences the outcome of household decision-making. Strategic behaviour between spouses centres on the non-transferable nature of health capital and the practice of gender discrimination. [26] The effect of India’s commonly practiced paternal-linked intergenerational household on bargaining power and household health capital investment is assumed to further disempower women.

There exist three broad cadres of allopathic outpatient healthcare providers in rural North India. Within the formal sector government MBBS (*GDr*) and private MBBS (*PDr*) operate. In the informal sector, unqualified (*jhola chhaap—JC*) providers abound. The meaning of the term ‘unqualified’ in this study is given by the Hindi term *jhola chhaap*. This Hindi term is commonly used to refer to unqualified healthcare providers and carries negative over-tones. Other non-allopathic healthcare providers operate too. In this study, all non-allopathic providers are groups into an ‘*Other*’ category.

Several demographic and socio-economic variables, plus a proxy for fever severity, are presented in this study. Age and Household Size are continuous variables. Binary variables are used to capture gender (1 = female) and religion (1 = muslim). Household income is reported in log form. Respondents’ literacy levels are aggregated into three categories: illiterate (no schooling), literate (some primary or secondary schooling) and high literate (completed at least High School). Respondents self-nominated their primary employment category: farmer, labourer, unpaid domestic work, and all other categories (market seller, shopkeeper, tradesperson, government employee, business person, unemployed and other). Healthcare provider characteristics are captured by patient-reported prices charged by healthcare providers (Indian Rupees—INR) and providers’ location relative to respondents’ residence (distance—kms). Duration of the fever is recorded via four categorical variables reflecting day intervals: 1–3 days, 4–6 days, 7–9 days and 10 and more days.

## Results

The distribution of healthcare provider consultations for a single episode of fever within the last 12-months is consistent across providers and by respondent gender. The frequency histograms for males and females—Fig 1, for each healthcare provider, show a bimodal distribution of visits for consultations greater than zero. The very low number of two visit treatment consultations is evident for males and females across all healthcare providers. The literature on the pattern of prescribing medicines over the course of fever treatment for a single episode of fever is limited for India. Based on preliminary qualitative observations made in preparation of the survey (see Figure A in S1 Supporting Information), it appeared a common practice among unqualified providers to dispense a small number of pills at any one consultation. This has the effect of requiring repeat visits to the same healthcare provider in order to receive more medicine.

The top panel in Fig 1 shows that female consumers have a higher frequency of one and four consultations to unqualified providers, compared to males. Consistently across providers females record a greater frequency of zero consultation for fever, compared to males.

The mean number of visits to a healthcare provider for a single episode of fever treatment is relatively stable across provider types. Discrete observations greater than five are truncated. The number of observations affected are small for each provider type (see Text A in S1 Supporting Information). Table 1 summarises the mean number of visits (μ), which are truncated, by gender and across the four outpatient provider groups–JC, GDr, PDr and Other (OT). On average, females consult unqualified healthcare providers 2.4 times per episode of fever and 1.0 time for Other non-MBBS providers. The qualified MBBS doctor rate of use is consistent at between 2.4 and 2.0 times, irrespective of government or private sector ownership and gender of patient. These mean number of visits is for a single provider. These figures don’t consider visits to other prover types for the same episode of fever.

**Table 1:** Mean number of visits to healthcare providers for a single episode of fever, by gender.

	JC		GDr		PDr		OT	
	Male	Female	Male	Female	Male	Female	Male	Female
μ rate	2.2	2.4	2.4	2.2	2.3	2.0	1.3	1.0
N	261	263	180	165	105	93	53	53

Note: JC = unqualified ‘doctors’; GDR = Government MBBS doctor; PDR = private MBBS doctor and OT = Other.

Table 2 presents the mean and categorical variable percentages for the 12-month recall sample used. The mean *price* for the 12-months recall group is INR 98.2. The percentage of consumers travelling either 0–4 km and 5–9 km were 41.9 per cent and 24.8 per cent. The values for the distance intervals 10–14 km, 15–19 km and 20+ km for each recall group were 12.1, 4.2 and 2.5 per cent. These descriptive statistics are consistent in comparison with those using a 14-day or less recall period.[27]

**Table 2:** Descriptive statistics of ≤ 12-month sample.

Variable	Definition	≤ 12-months
		Mean (se)/%
Age	Age in years (18 years and over)	39.6 (15.6)
Female (%)	Percentage of female respondents	51.1
Price	Mean price across all healthcare provider types in INR	98.2 (253.6)
lnhinc	Log of annual household Income	10.7 (0.7)
Hhsize	Number of family members in joint household	6.9 (3.1)
**Illness Duration (%)**		
Dur1	Percentage of respondents with a fever lasting 1–3 days	42.1
Dur2	Percentage of respondents with a fever lasting 4–6 days	31.6
Dur3	Percentage of respondents with a fever lasting 7–9 days	11.6
Dur4	Percentage of respondents with a fever lasting 10+ days	14.7
**Travel distance interval–healthcare provider (% of all providers)**		
D0	Percentage of respondents who accessed healthcare providers at home	1.0
D1	Percentage of respondents who travelled within the village	13.7
D2	Percentage of respondents who travelled 0–4 km	41.9
D3	Percentage of respondents who travelled 5–9 km	24.8
D4	Percentage of respondents who travelled 10–14 km	12.1
D5	Percentage of respondents who travelled 15–19 km	4.2
D6	Percentage of respondents who travelled 20+ km	2.5
Caste* (% of total sample, including Muslim respondents)		
Caste1	Percentage of respondents who are Brahmin	12.1
Caste2	Percentage of respondents who are Kshratrya	4.0
Caste3	Percentage of respondents who are Vaisya	36.9
Caste4	Percentage of respondents who are Shuda	22.0
Tribe	Percentage of respondents who are Tribal	3.5
Other	Percentage of respondents who are none of the above caste	21.3
**Literacy (%)**		
Illit	Percentage of respondents who self-reported being illiterate	45.3
Lit	Percentage of respondents who self-reported having completed some schooling less than completed High School	43.7
High Lit	Percentage of respondents who self-reported having completed at least Senior High School.	10.9
**Employment (%)**		
Job 1	Percentage of respondents who are classified as a Farmer	25.4
Job 2	Percentage of respondents who are classified as a labourer	24.5
Job 3	Percentage of respondents who are classified as a Market seller	0.5
Job 4	Percentage of respondents who are classified as a Shopkeeper	2.7
Job 5	Percentage of respondents who are classified as a Tradesperson	2.2
Job 6	Percentage of respondents who are classified as government employees	1.7
Job 7	Percentage of respondents who are classified as running own business	0.4
Job 8	Percentage of respondents who are classified as Unemployed	7.9
Job 9	Percentage of respondents who are classified as unpaid Domestic worker	29.7
Job 10	Percentage of respondents who are classified as in the “Other” category	5.0
**District (%)**		
DistA	Percentage of respondents in District A	46.5
CHC	Percentage of respondents in Village 1 which has a Community Health Centre	6.2
PHC1	Percentage of respondents in Village 2 which has a Primary Health Centre	15.9
DistB	Percentage of respondents in District B	30.0
DistC	Percentage of respondents in District C	23.6
PHC2	Percentage of respondents in Village 8 which has a Primary Health Centre	12.2

Note: Standard deviations are in parenthesis where appropriate.

* The Vedic caste-system contains four classes (varna): Brahman, Kshatriya, Vaishya and Shudra. Jaffrelot (2010) quotes the following famous myth allegory: ‘the Brahman (priest) was his mouth, his arm was made the [sic] Kshatriya (warrior), his thighs became the Vaishya and his feet the Shudra (servant) was made’.

Utilisation results are presented using pooled male and female responses for each provider type. The parameter estimation from the OP and ZIOP estimators is provided in S1 Supporting Information (see Table C in S1 Supporting Information) and account for village clustering in robust standard errors and the truncated visits greater than five. This clustering should control for expected village-based correlations between observed outcomes due to environmental factors affecting fever incidence.

The mean conditional marginal effects derived from the ZIOP model estimates are presented in Table 3. These results reveal that gender, religion, distance and price differences are instrumental in determining the pattern of utilisation of outpatient fever treatment. Other variables listed in Table 2 but not presented in Table 3 output results were not important variables in explaining the number of visits to healthcare providers for a single episode of fever. The marginal effect of *Price* on the likelihood of consulting a government and private MBBS provider, at least once, is consistently negative for government MBBS and positive for private MBBS. This is true at each value greater than zero, except for two visits. The negative marginal effect of *Price* on the likelihood of consulting a government MBBS one or more times suggests that consumers are price sensitive to the need to make informal payments. The positive *Price* marginal effect coefficient for one or more private MBBS provider visits indicates that for these doctors’ price acts as a marker of quality. This price signalling relates to both the medicines prescribed and the perceived quality of the consultation.

**Table 3:** Marginal effects from 12-month recall ZIOP model for each provider.

	JC			Gdr			Pdr				JC			Gdr			Pdr		
Variables	Co.Eff		p-value	Co.Eff		p-value	Co.Eff		p-value	Variables	Co.Eff		p-value	Co.Eff		p-value	Co.Eff		p-value
Y* = 0										Y* = 3									
Price	<-0.001		0.479	0.001	***	<0.001	0.001	***	0.001	Price	<0.001		0.479	<-0.001	***	<0.001	<0.001	***	<0.001
Lnhinc	0.007		0.763	0.045	**	0.015	0.002		0.754	Lnhinc	-0.003		0.763	-0.014	**	0.016	<0.001		0.773
Dur2 ^d^	-0.092	***	0.005	-0.057	**	0.047	0.006		0.842	Dur2 ^d^	0.034	***	0.006	0.018	**	0.049	-0.001		0.841
Dur3 ^d^	-0.048		0.294	-0.059		0.150	-0.076	*	0.060	Dur3 ^d^	0.018		0.301	0.019		0.154	0.012	*	0.051
Dur4 ^d^	-0.137	**	0.038	-0.141	**	0.036	-0.041		0.544	Dur4 ^d^	0.053	**	0.045	0.046	**	0.037	0.006		0.562
D1 ^d^	-0.174	***	<0.001		-			-		D1 ^d^	0.066	***	<0.001		-			-	
D2 ^d^	0.083	**	0.024		-		0.131	***	<0.001	D2 ^d^	-0.030	**	0.021		-		-0.018	***	0.001
D3 ^d^	0.331	***	<0.001		-		0.077	**	0.021	D3 ^d^	-0.107	***	<0.001		-		-0.011	**	0.011
D4 ^d^	0.353	***	<0.001		-		0.127	***	0.005	D4 ^d^	-0.108	***	<0.001		-		-0.016	***	0.001
Muslim ^d^	-0.107	***	0.005	0.071	**	0.014	-0.016		0.571	Muslim ^d^	0.040	***	0.006	-0.022	**	0.011	0.002		0.573
Female ^d^	-0.082	**	0.018	0.007		0.819	0.020		0.396	Female ^d^	0.030	**	0.018	-0.002		0.819	-0.003		0.418
Job1 ^d^	-0.091	**	0.045	-0.070	*	0.085		-		Job1 ^d^	0.034	**	0.048	0.022	*	0.084		-	
Job2 ^d^	-0.116	***	0.008	0.078	**	0.023		-		Job2 ^d^	0.044	**	0.010	-0.024	**	0.023		-	
Job9 ^d^	0.006		0.903	-0.044		0.293		-		Job9 ^d^	-0.002		0.903	0.014		0.295		-	
Y* = 1										Y* = 4									
Price	<0.001		0.480	<-0.001	***	<0.001	-0.001	***	<0.001	Price	<0.001		0.480	<-0.001	***	<0.001	<-0.001	***	<0.001
Lnhinc	-0.003		0.763	-0.018	**	0.013	-0.001		0.754	Lnhinc	-0.001		0.763	-0.007	**	0.026	<0.001		0.775
Dur2 ^d^	0.031	***	0.003	0.022	**	0.030	-0.004		0.842	Dur2 ^d^	0.017	***	0.009	0.009	*	0.073	<0.001		0.842
Dur3 ^d^	0.016		0.263	0.022		0.119	0.055	*	0.060	Dur3 ^d^	0.009		0.325	0.010		0.192	0.005	*	0.080
Dur4 ^d^	0.039	***	0.005	0.047	***	0.008	0.030		0.544	Dur4 ^d^	0.030	*	0.089	0.027	*	0.088	0.003		0.574
D1 ^d^	0.056	***	<0.001		-			-		D1 ^d^	0.034	***	<0.001		-			-	
D2 ^d^	-0.032	**	0.035		-		-0.103	***	<0.001	D2 ^d^	-0.014	**	0.016		-		-0.007	***	0.005
D3 ^d^	-0.155	***	<0.001		-		-0.059	**	0.019	D3 ^d^	-0.041	***	<0.001		-		-0.004	**	0.020
D4 ^d^	-0.177	***	<0.001		-		-0.102	***	0.005	D4 ^d^	-0.040	***	<0.001		-		-0.006	***	0.004
Muslim ^d^	0.034	***	0.002	-0.030	**	0.012	0.012		0.568	Muslim ^d^	0.021	**	0.013	-0.010	**	0.021	0.001		0.579
Female ^d^	0.029	**	0.018	-0.003		0.819	-0.015		0.395	Female ^d^	0.015	**	0.021	-0.001		0.819	-0.001		0.424
Job1 ^d^	0.030	**	0.031	0.027	*	0.063		-		Job1 ^d^	0.017	*	0.065	0.012		0.109		-	
Job2 ^d^	0.038	***	0.004	-0.033	**	0.020		-		Job2 ^d^	0.023	**	0.019	-0.012	**	0.038		-	
Job9 ^d^	-0.002		0.904	0.017		0.275		-		Job9 ^d^	-0.001		0.903	0.007		0.314		-	
Y* = 2										Y* = 5									
Price	<0.001		0.492	<-0.001		0.150	<-0.001		0.916	Price	<0.001		0.480	<-0.001	***	0.006	<-0.001	**	0.013
Lnhinc	<0.001		0.766	-0.003		0.175	<-0.001		0.876	Lnhinc	<0.001		0.763	-0.003	*	0.061	<-0.001		0.788
Dur2 ^d^	0.006	*	0.077	0.004		0.225	<-0.001		0.925	Dur2 ^d^	0.004	**	0.019	0.004		0.125	<0.001		0.840
Dur3 ^d^	0.003		0.340	0.004		0.286	0.001		0.916	Dur3 ^d^	0.002		0.348	0.004		0.239	0.003		0.127
Dur4 ^d^	0.008		0.105	0.009		0.213	<0.001		0.917	Dur4 ^d^	0.007		0.136	0.012		0.149	0.001		0.604
D1 ^d^	0.010	**	0.031		-			-		D1 ^d^	0.008	***	0.001		-			-	
D2 ^d^	-0.005	*	0.100		-		-0.001		0.915	D2 ^d^	-0.003	**	0.018		-		-0.003	*	0.067
D3 ^d^	-0.021	**	0.018		-		-0.001		0.916	D3 ^d^	-0.007	***	<0.001		-		-0.002	*	0.093
D4 ^d^	-0.022	**	0.017		-		-0.001		0.916	D4 ^d^	-0.006	***	<0.001		-		-0.002	*	0.073
Muslim ^d^	0.006	*	0.069	-0.005		0.214	<0.001		0.918	Muslim ^d^	0.005	**	0.025	-0.004	**	0.031	<0.001		0.585
Female ^d^	0.005	*	0.100	-0.001		0.820	<0.001		0.914	Female ^d^	0.003	**	0.026	<0.001		0.821	-0.001		0.462
Job1 ^d^	0.006		0.124	0.005		0.275		-		Job1 ^d^	0.004	*	0.090	0.005		0.154		-	
Job2 ^d^	0.007	*	0.068	-0.006		0.190		-		Job2 ^d^	0.005	**	0.037	-0.004	*	0.069		-	
Job9 ^d^	<0.001		0.904	0.003		0.389		-		Job9 ^d^	<0.001		0.903	0.003		0.348		-	

Note: Y* = N are the number of visits

d dummy variable

Statistical Significance

* 10%

** 5%

***1%

These results show that the number of patient visits indicates that patient behaviour is also important in determining repeat visits. This is particularly true for the marginalised groups: women and Muslims. Only for estimates of women consulting an unqualified provider is the binary *Female* variable positive and statistically significant at the 5 percent level. At all visit frequency for qualified providers the *Female* variable is not statistically significant. The marginal values indicate that Muslim are less likely to seek fever treatment from government MBBS providers. At the values one, three, four and five visits, the dummy variable for the marginal effect of Muslim identity on government MBBS provider utilisation is negative and statistically significant at the 5 percent level. The negative marginal effect estimates for *Price* and *Lnhinc* for qualified government providers are contrasting to those for unqualified providers.

A second set of ZIOP estimates are provided in Table 4. These estimates are run separately for male and female respondents for all consultations to unqualified *JC* providers. The estimated coefficients for the ZIOP model presented in Table 4 may be found in S1 Supporting Information (see Table D in S1 Supporting Information). The results presented in Table 4 show that the distance variables *D1*, *D2*, *D3*, *D4* for males are statistically significant at the 5 percent level. The estimates for *D1* (within village) is consistently positive and statistically significant at the 1 percent level across visit numbers 1, 3, 4, 5. The variables *D2—D4* are negative and variable in their significance below the 10 percent level. In contrast, the variable *D1* is consistently negative. These distance variables are relative to the *D0* (At Home).

**Table 4:** Marginal effects from 12-month recall ZIOP model for unqualified providers, by gender.

	Males			Females				Males			Females		
Variables	Co.Eff		p-value	Co.Eff		p-value	Variables	Co.Eff		p-value	Co.Eff		p-value
Y* = 0							Y* = 3						
Price	<0.001		0.814	<-0.001	***	<0.001	Price	<-0.001		0.814	<0.001	***	<0.001
Lnhinc	-0.030		0.472	0.012		0.724	Lnhinc	0.011		0.467	-0.005		0.724
Dur2 ^d^	0.024		0.613	-0.119	**	0.013	Dur2 ^d^	-0.009		0.611	0.047	**	0.016
Dur3 ^d^	0.096		0.136	0.282	***	<0.001	Dur3 ^d^	-0.033		0.116	-0.100	***	<0.001
Dur4 ^d^	0.118		0.165	0.054		0.480	Dur4 ^d^	-0.039		0.131	-0.021		0.470
D1 ^d^	-0.200	***	<0.001	0.087	*	0.061	D1 ^d^	0.074	***	0.001	-0.034	*	0.061
D2 ^d^	0.112	**	0.043	0.216	***	<0.001	D2 ^d^	-0.038	**	0.036	-0.080	***	<0.001
D3 ^d^	0.281	***	<0.001	0.380	***	<0.001	D3 ^d^	-0.089	***	<0.001	-0.131	***	<0.001
D4 ^d^	0.303	***	<0.001	0.405	***	<0.001	D4 ^d^	-0.091	***	<0.001	-0.135	***	<0.001
Muslim ^d^	-0.066		0.255	-0.156	***	0.004	Muslim ^d^	0.024		0.262	0.063	***	0.004
Job1 ^d^	0.076		0.193	0.092		0.103	Job1 ^d^	-0.027		0.184	-0.035	*	0.099
Job2 ^d^	0.014		0.837	0.177	***	<0.001	Job2 ^d^	-0.005		0.836	-0.067	***	<0.001
Job9 ^d^	0.042		0.452	0.239	***	<0.001	Job9 ^d^	-0.015		0.447	-0.090	***	<0.001
Y* = 1							Y* = 4						
Price	<-0.001		0.480	<0.001	***	<0.001	Price	<-0.001		0.814	<0.001	***	<0.001
Lnhinc	0.011		0.763	-0.004		0.726	Lnhinc	0.004		0.466	-0.002		0.724
Dur2 ^d^	-0.009		0.003	0.038	***	0.006	Dur2 ^d^	-0.003		0.608	0.017	**	0.030
Dur3 ^d^	-0.040		0.263	-0.125	***	<0.001	Dur3 ^d^	-0.012		0.101	-0.028	***	<0.001
Dur4 ^d^	-0.051		0.005	-0.020		0.508	Dur4 ^d^	-0.014	*	0.099	-0.007		0.448
D1 ^d^	0.065	***	<0.001	-0.031	*	0.069	D1 ^d^	0.033	***	0.008	-0.011	*	0.065
D2 ^d^	-0.046	*	0.035	-0.087	***	<0.001	D2 ^d^	-0.014	**	0.035	-0.024	***	<0.001
D3 ^d^	-0.132	***	<0.001	-0.173	***	<0.001	D3 ^d^	-0.030	***	<0.001	-0.037	***	<0.001
D4 ^d^	-0.152	***	<0.001	-0.193	***	<0.001	D4 ^d^	-0.029	***	<0.001	-0.037	***	<0.001
Muslim ^d^	0.023		0.002	0.046	***	0.001	Muslim ^d^	0.010		0.278	0.024	**	0.020
Job1 ^d^	-0.030		0.031	-0.034		0.121	Job1 ^d^	-0.010		0.179	-0.011	*	0.094
Job2 ^d^	-0.005		0.004	-0.068	***	0.001	Job2 ^d^	-0.002		0.835	-0.021	***	0.001
Job9 ^d^	-0.016		0.904	-0.091	***	<0.001	Job9 ^d^	-0.006		0.440	-0.029	***	<0.001
Y* = 2							Y* = 5						
Price	<-0.001		0.816	<0.001		0.161	Price	<-0.001		0.816	<0.001	***	<0.001
Lnhinc	0.002		0.519	-0.001		0.730	Lnhinc	0.002		0.458	-0.001		0.726
Dur2 ^d^	-0.002		0.636	0.006		0.212	Dur2 ^d^	-0.001		0.608	0.011	*	0.062
Dur3 ^d^	-0.007		0.294	-0.015		0.157	Dur3 ^d^	-0.004	*	0.093	-0.014	***	<0.001
Dur4 ^d^	-0.009		0.321	-0.003		0.535	Dur4 ^d^	-0.005	*	0.078	-0.004		0.427
D1 ^d^	0.014		0.162	-0.005		0.248	D1 ^d^	0.013	**	0.021	-0.007	*	0.081
D2 ^d^	-0.008		0.222	-0.012		0.166	D2 ^d^	-0.005	*	0.055	-0.013	***	0.001
D3 ^d^	-0.021		0.133	-0.020		0.161	D3 ^d^	-0.009	***	0.003	-0.019	***	<0.001
D4 ^d^	-0.022		0.125	-0.022		0.157	D4 ^d^	-0.009	***	0.004	-0.019	***	<0.001
Muslim ^d^	0.005		0.359	0.008		0.205	Muslim ^d^	0.004		0.338	0.016	**	0.030
Job1 ^d^	-0.006		0.340	-0.005		0.282	Job1 ^d^	-0.004		0.173	-0.007	*	0.099
Job2 ^d^	-0.001		0.840	-0.009		0.186	Job2 ^d^	-0.001		0.833	-0.012	***	0.003
Job9 ^d^	-0.003		0.503	-0.013		0.173	Job9 ^d^	-0.002		0.441	-0.016	***	0.001

Note: Y* = N are the number of visits

d dummy variable

Statistical Significance

* 10%

** 5%

***1%

The marginal effects estimates for *Price* are contrasting between men and women in consulting unqualified providers. The *Price* estimates are consistently positive and statistically significant at the 1 percent level for visits 1, 3, 4 and 5. The corresponding estimates for men are highly insignificant. The *Lnhinc* variable is insignificant for both men and women consulting unqualified providers. However, the employment category and variable *Unpaid* is negative and statistically significant at the 1 percent level. This may suggest that women’s independent access to household income is a factor affecting choice of provider and number of number of visits.

The marginal effect coefficient for the *Muslim* identity dummy variable shows that Muslim women are also more likely to consult unqualified providers than their male counterparts. The *Muslim* female coefficients are positive and statistically significant at the 5 percent level. The reason why there exists a tendency for Muslim consumers not to consult government MBBS providers, as evidenced in Table 3 and Table 4, is unclear. The effect of some dimension of consumer *trust*, and lack of *trust* in individual government doctors or the institution, is a possible explanation.

## Conclusion

This study indicates that women in north India are less likely to access outpatient fever care. Further, when women do access outpatient care for mild-sever fever they are more likely to consult with low quality unqualified outpatient healthcare providers. The analysis indicates that men have greater access to healthcare resources compared to women. While Hindu and Muslim women are more likely to consult unqualified providers for episodes of fever women who identify as primarily performing unpaid domestic work are less likely to consult the same unqualified providers. The conclusion that women have limited access to healthcare resources, relative to men, is further supported by the consistent finding that women are less likely to consult a private qualified MBBS provider.

Muslim respondents have an increased likelihood of consulting unqualified healthcare providers and a reduced likelihood of consulting qualified government providers. The strong marginal effects estimates for Muslim women consulting unqualified providers for episodes of fever appear to drive the disaggregated Muslim results. Insufficient power limits the available insights into whether there exists a gendered effect related to Muslim’s preferences for qualified government outpatient receives in rural north India.

The causal factors that drive the observed distribution of repeat visits across all provider types is unclear. While the lack of a single repeat visit for fever treatment (i.e. two consultations in total) is surprising, the current data does not allow for explanatory analysis of this data. The lack of data on healthcare provider behaviour limits the current study’s ability to explain this distribution.

As is common among studies replying on self-reported recall survey data, the results presented are subject to recall bias. However, it is assumed that such effects are equally present across all respondents. Therefore, the effect of any such biases when comparing estimates within a model has no net effect. The use of a relatively small sample limits the generalisability of results. Despite the belief that the sample is representative across the three sample districts. Care should be taken in generalising beyond the sample.

The results of the current study confirm those of other studies that women’s utilisation of outpatient healthcare for communicable illnesses in LMICs is often less than men.[28, 29] One likely interpretation of the results presented here is that demand is less due to woman’s reduced access to household financial resources. In the present study, women have an increased likelihood of consulting an unqualified provider, relative to consulting qualified providers in the public or private sectors. The widespread practice of public sector patients paying informal payments to government providers causes the total cost of accessing public sector services being greater than the sum of travel costs and INR 1 administration fee.[12] As a consequence, the cost of accessing government MBBS providers is likely greater than locally available unqualified providers.[16] As a consequence of women’s reliance on unqualified providers to treat fever symptoms, expected outcomes of regulatory reform concerning the informal sector will disproportionately affect women and Muslims in rural North India.

In the context of regulatory reform of healthcare in LMICs and, in particular, the role of the private sector within systems of universal health coverage, how the informal sector should be managed is an important policy question.[1] The role that the unqualified outpatient healthcare sector plays in providing fever treatments to women and Muslim communities is important. The localised results presented in this study indicate that marginalised groups disproportionately rely on unqualified providers who operate in the informal healthcare sector. These results are consistent with the healthcare outcomes expected when applying intra-household bargaining theory to explain healthcare utilisation.[22, 24] The more detailed data presented in this study, concerning alternative healthcare providers and the timing of patients’ accessing outpatient healthcare, provides new insights into how heterogeneous communities engage with healthcare systems in LMICs. As a result, healthcare regulatory reform in LMICs aimed at eliminating all forms of care offered by unqualified providers is expected to regressively affect marginalised communities.

## Supporting information

S1 Supporting Information Text A. Table A. Table B. Text B. Table C. Table D. Table E. Table C. Figure A. Summary of provider survey results.
(DOCX)
