# Supplementary material for: Informal healthcare sector and marginalized groups: Repeat visits in rural North India
Source: PLoS One. 2018 Jul 6;13(7):e0199380. doi: 10.1371/journal.pone.0199380 (PMC6034796; doi:10.1371/journal.pone.0199380)

**Supplementary Material**

**Text A**

The pattern of patient visits to providers shows no signs of statistical difference across shorter recall periods. S1 Table presents the percentage distribution of healthcare provider visits by recall period. The table contains the following: column (1) lists the number of visits recorded as a value between one – five, columns (2)-(5) list the percentage of observed healthcare provider visits for a single fever episode for independent recall periods, ≤ 14 days, 15-30 days, 2-6 months, 7-12 months; column (6) is the weighted average of the percentage in the preceding four columns; and column (7) gives the *p*-values for the null hypothesis that values in the ≤ 14-day recall are the same as those in the 7-12 month recall period. The *p*-values in column (7) indicate that there is no statistically significant difference between the percentage of healthcare provider visits at each discrete interval, in the range one to five.

Table A. Distribution of visits to healthcare providers across recall periods ≤ 14-days to 7-12 months

| Number of Visits | ≤ 14-days | 15-30 days | 2-6 months | 7-12 months | Average * | 𝝌2 test p-values |
| --- | --- | --- | --- | --- | --- | --- |
|  |  |  |  |  |  |  |
| (1) | (2) | (3) | (4) | (5) | (6) | (7) = (2) vs (5) |
| 1 | 49.2 | 53.0 | 51.6 | 48.5 | **50.7** | 0.897 |
| 2 | 4.4 | 5.2 | 4.0 | 7.6 | **5.0** | 0.142 |
| 3 | 25.6 | 25.9 | 31.3 | 29.2 | **27.8** | 0.391 |
| 4 | 14.8 | 12.4 | 10.7 | 10.5 | **12.4** | 0.188 |
| 5 | 3.4 | 2.8 | 1.2 | 4.1 | **2.8** | 0.686 |
| > 5 | 2.7 | 0.8 | 1.2 | 0.0 | **1.3** | 0.030 |
| n | 297 | 251 | 252 | 171 | 971 |  |

The dependent variable in count and ordered probit models are discrete values. A plausible explanation for the low frequency of two visits, as highlighted in S1 Table, is the confounding of the number of visits by severity of fever. S2 Table provides the frequency distribution of visits to i) unqualified providers (JC), ii) government MBBS providers (GDr), and iii) private MBBS provider (PDr) across fever duration intervals. Controlling for severity of fever, the frequency data in Table S1.2 demonstrates that there is a uniform absence in the percentage of respondents making two visits to all health provider types. The percentage distribution of zero visits is the largest observed outcome across each provider type. Naturally, as the market share of any provider type declines, within the 12-month recall period, the resulting percentage of zero visits, all other things being equal, increases. This explains the first cause of zero visits. Those consumers who have been sick in the past 12 months have initially sought fever treatment from another provider. The second explanation for the high percentage of zeros observed is that respondents did not consider themselves as having fever symptoms of sufficient severity to warrant seeking treatment.

Table B. Distribution of visits to healthcare providers in 12-month recall period, by provider type and fever duration

| Visits # | 1-3 days | | | 4-6 days | | | 7-9 days | | | 10-12 days | | | 13+ days | | |
| --- | --- | --- | --- | --- | --- | --- | --- | --- | --- | --- | --- | --- | --- | --- | --- |
|  | **JC** | **Gdr** | **Pdr** | **JC** | **Gdr** | **Pdr** | **JC** | **Gdr** | **Pdr** | **JC** | **Gdr** | **Pdr** | **JC** | **Gdr** | **Pdr** |
| 0 | 22.6 | 32.6 | 35.3 | 17.8 | 21.9 | 27.1 | 8.1 | 8.5 | 9.2 | 3.2 | 3.2 | 3.8 | 9.5 | 9.8 | 10.2 |
| 1 | 10.9 | 3.1 | 4.2 | 5.1 | 4.0 | 2.1 | 0.9 | 1.8 | 0.9 | 0.3 | 0.7 | 0.2 | 0.9 | 1.2 | 0.9 |
| 2 | 1.1 | 0.7 | 0.1 | 0.7 | 0.3 | 0.0 | 0.3 | 0.4 | 0.1 | 0 | 0.1 | 0.0 | 0.2 | 0.2 | 0.0 |
| 3 | 4.7 | 2.3 | 0.7 | 5.0 | 3.1 | 1.2 | 1.5 | 0.6 | 0.7 | 0.4 | 0.3 | 0.5 | 0.3 | 0.9 | 0.3 |
| 4 | 1.2 | 1.6 | 0.3 | 1.8 | 1.0 | 0.1 | 0.7 | 0.3 | 0.5 | 0.4 | 0.3 | 0.1 | 1.1 | 0.1 | 0.3 |
| 5-n* | 0.0 | 0.3 | 0.1 | 0.3 | 0.3 | 0.2 | 0.2 | 0.2 | 0.3 | 0.3 | 0.2 | 0.1 | 0.4 | 0.3 | 0.6 |
| * n(max) values: JC=11, Gdr=11, Pdr=13 | | | |  |  |  |  |  |  |  |  |  |  |  |  |

**Text B**

The utilisation decisions of adults are modelled according to deterministic random utility model functions. The splitting equation is the binary model of self-reporting a fever illness and is provided in equation (1). Equation (1) is provided below:

*P(Fever^12-months^=1) = β_0_ + β_1_ Illit + β_2_ Age* + *β_3_ Rational+ β_4_ Lnhinc + u,*

(1)

where *Illit* denotes self-reporting of no schooling, *Age* is the represents those over the age of 18 years, *Rational* is a binary variable indicating whether the respondent correctly identified a dominant choice alternative in a Discrete Choice Experiment task given prior to the survey, and *Lnhinc* is the log of household income.

The reduced form utility function used in the ZIOP model is given below:

$U=U(X,H,Z)$, (2)

where **X**, **H** and **Z** are vectors for consumer characteristics, health status and provider characteristics. The models 1 and 2 presented in this study use additive utility functions.

**Table C. Zero-inflated Ordered Probit coefficient estimates by provider type – clustered and truncated**

|  | JC | | Gdr | | Pdr | |
| --- | --- | --- | --- | --- | --- | --- |
|  | Co.Eff |  | Co.Eff |  | Co.Eff |  |
| Price | <0.001 |  | -0.004 | *** | -0.006 | *** |
| Lnhinc | -0.019 |  | -0.170 | ** | -0.007 |  |
| Dur2 ^d^ | 0.243 | *** | 0.208 | ** | -0.022 |  |
| Dur3 ^d^ | 0.128 |  | 0.206 |  | 0.268 | ** |
| Dur4 ^d^ | 0.354 | ** | 0.452 | ** | 0.149 |  |
| D1 ^d^ | 0.461 | *** | - | | - | |
| D2 ^d^ | -0.232 | ** | - | | -0.618 | *** |
| D3 ^d^ | -1.151 | *** | - | | -0.335 | ** |
| D4 ^d^ | -1.407 | *** | - | | -0.654 | ** |
| Muslim ^d^ | 0.280 | *** | -0.291 | ** | -0.077 |  |
| Female ^d^ | 0.221 | ** | -0.026 |  | 0.061 |  |
| Job1 ^d^ | 0.240 | ** | 0.249 | * | - | |
| Job2 ^d^ | 0.305 | *** | -0.316 | ** | - | |
| Job9 ^d^ | -0.015 |  | 0.160 |  | - | |
| Constant | -0.396 |  | 1.316 | * | - | |
| Note: ^d^ dummy variable | |  |  |  |  |  |
| Statistical Significance: * 10%, ** 5%, ***1% | | | |  |  |  |

**Table D. Zero-inflated Ordered Probit coefficient estimates for unqualified provider by gender – clustered and truncated**

|  | Males | |  | Females | |
| --- | --- | --- | --- | --- | --- |
| Variables | Co.Eff |  |  | Co.Eff |  |
| Price | <-0.001 |  |  | 0.001 | *** |
| Lnhinc | 0.080 |  |  | -0.032 |  |
| Dur2 ^d^ | -0.065 |  |  | 0.326 | ** |
| Dur3 ^d^ | -0.264 |  |  | -1.028 | *** |
| Dur4 ^d^ | -0.332 |  |  | -0.158 |  |
| D1 ^d^ | 0.521 | *** |  | -0.248 | * |
| D2 ^d^ | -0.308 | * |  | -0.686 | *** |
| D3 ^d^ | -0.879 | *** |  | -1.658 | *** |
| D4 ^d^ | -1.023 | *** |  | -2.682 | *** |
| Muslim ^d^ | 0.172 |  |  | 0.418 | *** |
| Job1 ^d^ | -0.205 |  |  | -0.265 |  |
| Job2 ^d^ | -0.038 |  |  | -0.536 | *** |
| Job9 ^d^ | -0.112 |  |  | -0.732 | *** |
| Constant | -0.921 |  |  | 0.795 |  |
|  |  |  |  |  |  |
| Mu(1) | 0.692 | *** |  | 0.56282 | *** |
| Mu(2) | 0.809 | *** |  | 0.64029 | *** |
| Mu(3) | 1.510 | *** |  | 1.35564 | *** |
| Mu(4) | 2.127 | *** |  | 1.83677 | *** |
| Note: ^d^ dummy variable | |  |  |  |  |
| Statistical Significance: * 10%, ** 5%, ***1% | | | | |  |

The results, presented in S5 Table, show that within model 1 the ZIOP estimates provide relatively mixed good-of-fit performance, compared to an OP, across the three healthcare provider alternatives. The coefficients for the four boundary parameters are all statistically significant at the one percent level for the OP and ZIOP models across the three data sets, except for the OP modelling private MBBS visits. The better performance of the ZIOP model in providing statistically significant boundary parameters across all healthcare providers justified its use. Further Goodness-of-Fit measures are provided in S5 Table.

Table E. Goodness-of-fit measures for ordered probit and zero-inflated ordered probit

|  | JC | | | | | | | | | | | | | | | | | | Gdr | | | | | | | | | | | | | | | | | | Pdr | | | | | | | | | | | | | | | | |
| --- | --- | --- | --- | --- | --- | --- | --- | --- | --- | --- | --- | --- | --- | --- | --- | --- | --- | --- | --- | --- | --- | --- | --- | --- | --- | --- | --- | --- | --- | --- | --- | --- | --- | --- | --- | --- | --- | --- | --- | --- | --- | --- | --- | --- | --- | --- | --- | --- | --- | --- | --- | --- | --- |
|  | OP | | | | | | ZIOP | | | | | | | | | | | | OP | | | | | | ZIOP | | | | | | | | | | | | OP | | | | | | | | | ZIOP | | | | | | | |
| 𝝁1 | 0.629 | | *** | | | <0.001 | | 0.640 | | *** | | | | | <0.001 | | | | 0.451 | | *** | | <0.001 | | | 0.453 | | *** | | | | | <0.001 | | | | 0.521 | | |  | | 0.152 | | | | 0.977 | *** | | | <0.001 | | | |
| 𝝁2 | 0.723 | | *** | | | <0.001 | | 0.735 | | *** | | | | | <0.001 | | | | 0.541 | | *** | | <0.001 | | | 0.543 | | *** | | | | | <0.001 | | | | 0.537 | | |  | | 0.147 | | | | 1.001 | *** | | | <0.001 | | | |
| 𝝁3 | 1.409 | | *** | | | <0.001 | | 1.427 | | *** | | | | | <0.001 | | | | 1.073 | | *** | | <0.001 | | | 1.074 | | *** | | | | | <0.001 | | | | 0.986 | | | *** | | 0.004 | | | | 1.459 | *** | | | <0.001 | | | |
| 𝝁4 | 2.218 | | *** | | | <0.001 | | 2.236 | | *** | | | | | <0.001 | | | | 1.655 | | *** | | <0.001 | | | 1.654 | | *** | | | | | <0.001 | | | | 1.306 | | | *** | | 0.001 | | | | 1.911 | *** | | | <0.001 | | | |
| LL |  | | | -1219.1 | | |  | | | | | -1216.1 | | | | | | |  | | | -945.22 | | |  | | | | | -945.14 | | | | | | |  | | | | -636.35 | | | | |  | | | -611.81 | | | | |
| AIC |  | 2476.2 | | | | |  | |  | | | | | | 2482.2 | | | |  | | 1920.4 | | | |  | | | |  | | | 1930.3 | | | | |  | | 1300.7 | | | | | | |  | 1259.6 | | | | | |  |
| BIC |  | 2537.5 | | | | |  | |  | | | | | | 2562.9 | | | |  | | 1968.9 | | | |  | | | |  | | | 1994.9 | | | | |  | | 1345.9 | | | | | | |  | 1317.7 | | | | | |  |
| LR versus OP  Vuong versus OP | | | | |  | | | | | |  | | | 12.11 | | | | |  | | |  |  | | | |  | | | |  | | | 5.25 | | |  | | | | | |  |  | | |  | | | |  | 25.38 |  |
|  |  |  |  |  |  | | | | | |  | | |  | | 2.6 | | |  | | |  |  | | | |  | | | |  | | | 2.04 | | |  | | | | | |  |  | | |  | | | |  | 3.29 |  |
| Statistical Significance: * 10%, ** 5%, ***1% | | | | | | | | | | | | |  | | | |  |  | |  | | |  |  | | | | |  | | | | | |  |  | |  | | | | | |  |  | | |  | | |  |  |  |

**Text C**

A multi-stage clustering sampling frame was used in Stages One and Two of the data collection. Stage One included 48 surveys with consumers, healthcare providers and local level key informants (Community Health Workers – *ASHAs* and Village Leaders – *pradhans*). Stage Two encompassed the delivery of the Revealed Preference (RP) surveys. An important constraint surrounding the data collection process was the need to control for potential seasonal effects associated with fever treatment demand. The monsoon season is expected to generate a high level of fever treatment demand in north India. Stage One data was collected during April and May in the dry-season of 2012. Stage Two data was collected during the north Indian monsoon season, which usually runs from mid-June to mid-September. However, in 2012 the monsoon onset was delayed by three-four weeks in north India. As a result, the average rainfall during the first 2-months of the season was below average. This contracted the duration of the monsoon and limited the window of opportunity. However, Stage Two data was still collected during the monsoon.

*Sampling frame*

Sampling of village households, within all villages except Village Two (Fatehpur), systematically covered all geographic sections in a quasi-random process. Maps of the villages were not available, so local knowledge of the village was drawn upon to ensure that enumerators sampled evenly across the whole village. This approximate even sampling across any given village was important as many villages were informally divided according to religion and caste. Enumerators selected the households and individuals to survey. As a check on the micro-level sampling by enumerators, sampling profiles for each enumerator of their survey respondents was monitored during each day of data collection. This monitoring of sampling profiles included consideration of mean age and gender proportions. In this light, the sampling of individuals conformed to a quota method. Village Two was the first village sampled. The village pradhan organised the recruitment of villagers according to our representative sample request.

*Choice set creation*

A survey of providers was also completed as part of Stage One of the study to help verify the range of provider characteristics available in the local outpatient market. Village level healthcare providers were identified by local key informants. S1 Fig provides a summary of the providers surveys and their attributes.

Broad uniformity among surveyed unqualified is evident from S1 Fig: average stated prices range between 40 – 60 rupees for a single visit, a margin of 10 rupees is added to the cost of medicine by providers, word-of-mouth recommendation were widely seen to be the main mechanism to grow one’s business and no direct advertising was employed by unqualified providers. All providers cited that fever symptoms were one of the leading complaints by patients. Based on the survey result of healthcare providers it is assumed that in the treatment of mild-severe fevers the attributes of unqualified providers were homogeneous.

**Figure A. Summary of provider survey results**


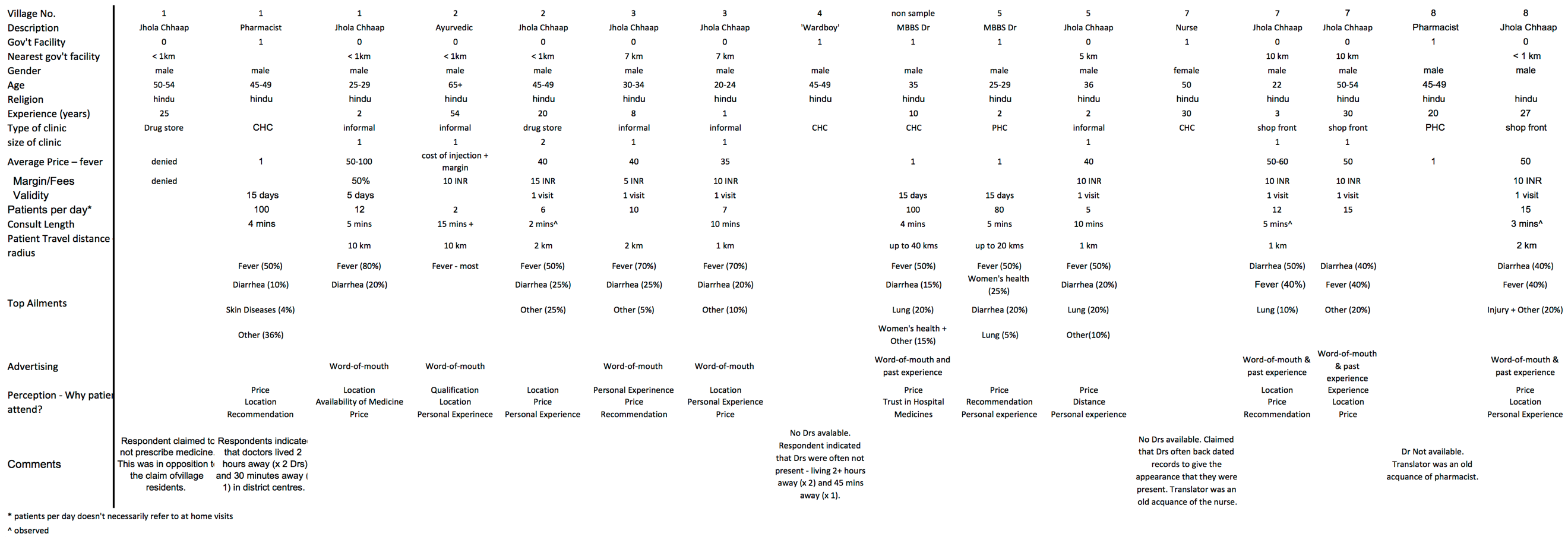

Supplement: S1 Supporting Information — Text A. Table A. Table B. Text B. Table C. Table D. Table E. Table C. Figure A. Summary of provider survey results. (DOCX) [file pone.0199380.s001.docx]
